# Supplementary figures and images for: Unraveling the Matrix: Proteomic Profiling Reveals Stromal ECM Dysregulation in Severe Early-Onset Fetal Growth Restriction
Source: Int J Mol Sci. 2025 Nov 19;26(22):11179. doi: 10.3390/ijms262211179 (PMC12653012; doi:10.3390/ijms262211179)

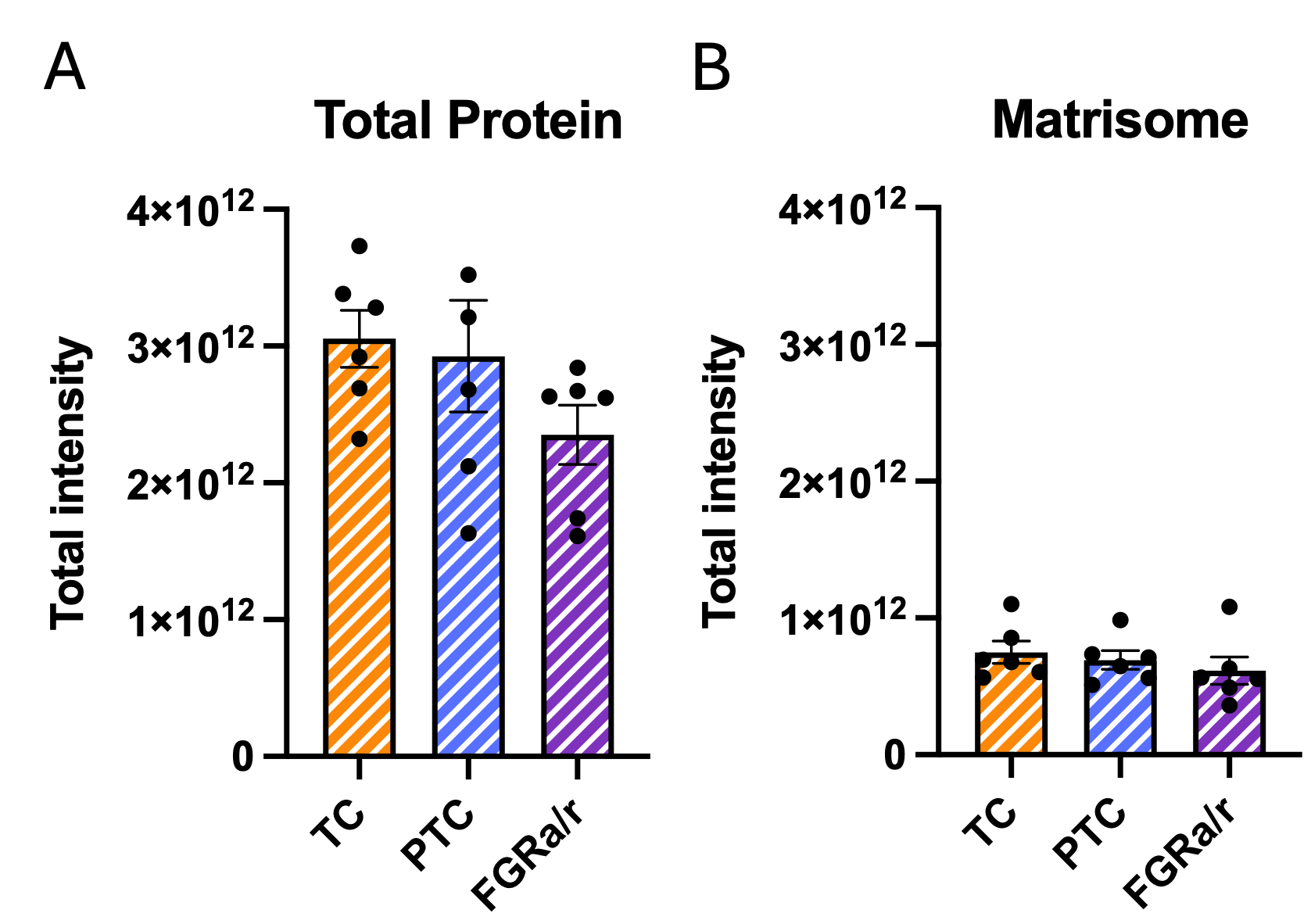

Supplement: Supplementary file 1 [file ijms-26-11179-s001.zip › Supplemental Figure S1.png]

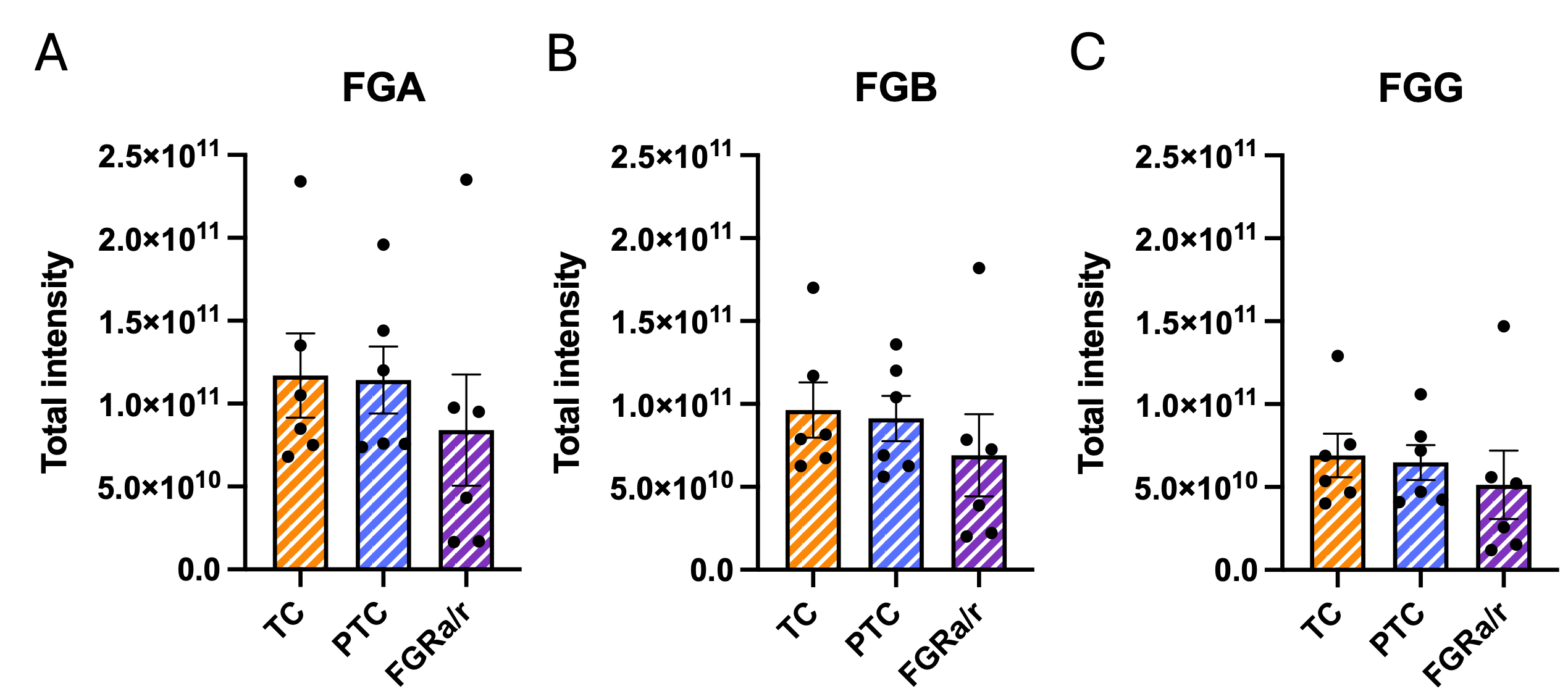

Supplement: Supplementary file 1 [file ijms-26-11179-s001.zip › Supplemental Figure S2.png]

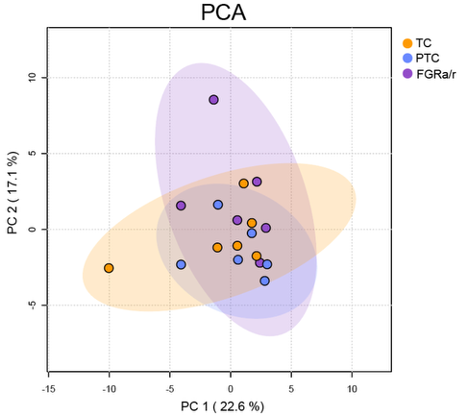

Supplement: Supplementary file 1 [file ijms-26-11179-s001.zip › Supplemental Figure S3.png]

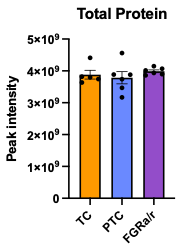

Supplement: Supplementary file 1 [file ijms-26-11179-s001.zip › Supplemental Figure S4.png]

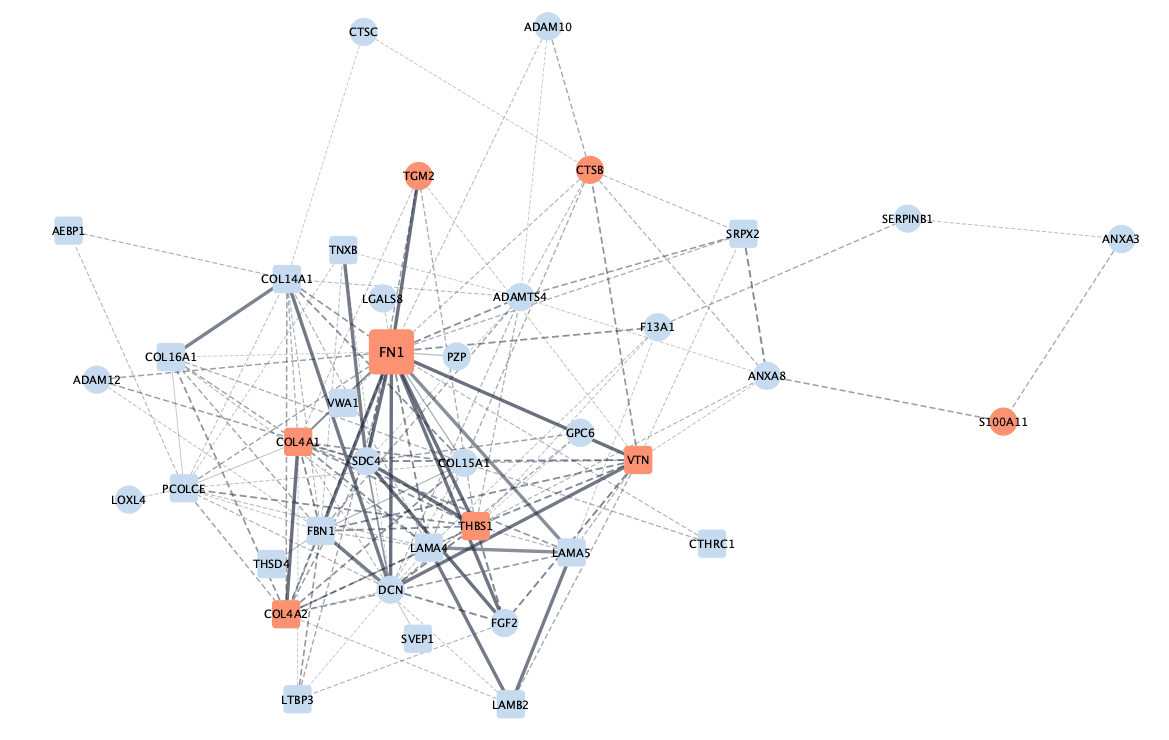

Supplement: Supplementary file 1 [file ijms-26-11179-s001.zip › Supplemental Figure S5.png]
